# Cotton disease identification method based on pruning

**DOI:** 10.3389/fpls.2022.1038791

**Published:** 2022-12-14

**Authors:** Dongqin Zhu, Quan Feng, Jianhua Zhang, Wanxia Yang

**Affiliations:** ^1^ School of Mechanical and Electrical Engineering, Gansu Agricultural University, Lanzhou, China; ^2^ Agricultural Information Institute, Chinese Academy of Agricultural Sciences, Beijing, China; ^3^ National Nanfan Research Institute, Chinese Academy of Agricultural Sciences, Sanya, China

**Keywords:** convolutional neural network, pruning, cotton diseases, transfer learning, compact model

## Abstract

Deep convolutional neural networks (DCNN) have shown promising performance in plant disease recognition. However, these networks cannot be deployed on resource-limited smart devices due to their vast parameters and computations. To address the issue of deployability when developing cotton disease identification applications for mobile/smart devices, we compress the disease recognition models employing the pruning algorithm. The algorithm uses the γ coefficient in the Batch Normalization layer to prune the channels to realize the compression of DCNN. To further improve the accuracy of the model, we suggest two strategies in combination with transfer learning: compression after transfer learning or transfer learning after compression. In our experiments, the source dataset is famous PlantVillage while the target dataset is the cotton disease image set which contains images collected from the Internet and taken from the fields. We select VGG16, ResNet164 and DenseNet40 as compressed models for comparison. The experimental results show that transfer learning after compression overall surpass its counterpart. When compression rate is set to 80% the accuracies of compressed version of VGG16, ResNet164 and DenseNet40 are 90.77%, 96.31% and 97.23%, respectively, and the parameters are only 0.30M, 0.43M and 0.26M, respectively. Among the compressed models, DenseNet40 has the highest accuracy and the smallest parameters. The best model (DenseNet40-80%-T) is pruned 75.70% of the parameters and cut off 65.52% of the computations, with the model size being only 2.2 MB. Compared with the version of compression after transfer learning, the accuracy of the model is improved by 0.74%. We further develop a cotton disease recognition APP on the Android platform based on the model and on the test phone, the average time to identify a single image is just 87ms.

## Introduction

Plant protection, especially crop protection against plant diseases, plays a critical role in meeting the growing demand for crop quality and quantity. In the 21st century, the issue of protecting crops from yield losses due to disease remains challenging. Worldwide, it is estimated that 20-40% of crop yield is lost due to pests and diseases (Savary et al., 2019). The loss of staple cereals (rice, wheat, corn) and vegetable crops (potatoes and sweet potatoes) directly affects food security and nutrition, while the loss of core commodity crops such as cotton has a significant impact on household livelihoods and the national economy. Plant diseases are an essential factor in the severe decline in the quality and quantity of agricultural products. Therefore, early detection and diagnosis of the diseases are key to reducing losses. At present, many developing countries identify diseases through visual observation (Chen et al., 2020), which requires disease detection experts with a lot of practical knowledge in the field. However, 80% of the world’s food is produced by individual farmers (Lu et al., 2021), and it is difficult for most farmers to correctly identify the category of crop diseases.

Cotton is a significant cash crop (Khan et al., 2020) and a vital raw material for the textile industry, which plays a critical part in the world. The vast distribution of cotton areas in China and the great differences in natural conditions have resulted in a wide range of cotton diseases. There are more than 80 kinds of cotton diseases recorded, of which about 20 are the most common. Cotton diseases annually cause significant losses in the yield and quality, especially fusarium wilt and verticillium wilt. If we can observe these diseases in time and give specific treatment measures, these diseases will be controlled. Improving disease control methods is one of the initiatives implemented to solve these issues. Disease identification methods should be cheap and easy to use for farmers. With the development of communication networks, smart phones have become very popular in rural areas, so disease identification based on smart phones is very promising. It is worth mentioning that disease identification methods of plant pathogens, including molecular biotechnologies such as DNA, RNA and protein are fast and accurate (Sapre et al., 2021). However, the preparation of diagnostic kits and their application require more expensive instruments and professional technical support. Hence, it is difficult to be applied in the field outside the laboratory in the short term.

In the past few years, image classification in computer vision has been greatly developed, especially the emergence of deep convolutional neural networks (DCNN), which have greatly improved the accuracy of object recognition. Currently, many convolutional neural networks with superior performance have been proposed, including AlexNet (Krizhevsky et al., 2017), VGG (Simonyan and Zisserman, 2014), GoogLeNet (Szegedy et al., 2015), ResNet (He et al., 2016) and DenseNet (Huang et al., 2017). These networks have been successfully applied in the agricultural field, such as plant disease identification (Bharathi, 2020), plant species identification (Ghazi et al., 2017), weeds classification (Hoang Trong et al., 2020) and fruit detection (Vasconez et al., 2020). With the help of DCNN, image-based plant disease identification becomes more accurate, fast and easy to use (Kamilaris and Prenafeta-Boldú, 2018; Liu and Wang, 2021; Dhaka et al., 2021). Mohanty et al. (2016) used PlantVillage to train AlexNet and GoogLeNet to identify diseased and healthy leaves of 14 species of plants. Their trained model achieved 99.35% accuracy on the testing set and evaluated the applicability of a DCNN to classification problems. Extending the work of Johannes et al. (2017); Picon et al. (2018) adopted an adaptive algorithm of deep residual networks to detect multiple plant diseases collected in natural environments, achieving a balanced accuracy of 0.87. Aiming at the problem of multiple parameters and single feature scale in AlexNet, Zhang et al. (2019) proposed a global pooling dilated convolutional neural network, which combined the advantages of global pooling and dilated convolution, and can effectively identify cucumber diseases. Chen et al. (2020) used VGG with Inception module trained on ImageNet dataset as a pre-training model and performed transfer learning on the public datasets and the self-built datasets, respectively. Experimental results showed that the proposed method achieves substantial improvement over other state-of-the-art methods. Kundu et al. (2020) experimented with eight different deep learning models on the public dataset of the bell pepper. Their experimental results showed that the DenseNet model outperforms several other models in identifying sweet pepper diseases. Mi et al. (2020) proposed a new convolutional neural network C-DenseNet which embedded Convolutional Block Attention Module into the DenseNet network to grade wheat stripe rust, which achieved a testing accuracy of 97.99%, higher than the original DenseNet (92.53%) and ResNet (73.43%). Jiang et al. (2021) used VGG16 to identify the diseases in rice and wheat leaves with an overall accuracy of 97.22% and 98.75%, respectively. Collecting large datasets to train these networks is still a daunting task, but many studies have demonstrated the feasibility of deep learning in disease areas, especially deep transfer learning (Sladojevic et al., 2016; Ghazi et al., 2017; Hassan et al., 2021). Although CNN and its variants have shown superior performance in the field of disease identification, these models have a large number of parameters and computations, which are difficult to deploy on some type of target hardware such as mobile or edge devices. In addition, in numerous disease identification studies, they are rarely involved in cotton diseases.

The application of deep learning technology in disease identification is inseparable from the development of convolutional neural networks. From AlexNet with only 8 layers in the beginning, to VGG19 with 19 layers later, to ResNet breaking through 100 layers for the first time, its development is attributed to various factors, including the introduction of a powerful computing system and Graphics Processing Unit (GPU), increased memory and hard disk capacity (Hou et al., 2018). Deep learning is impractical on low-memory and low-energy devices due to the size of networks. The success of many large networks almost depends on GPU. However, with the proliferation of smartphones, mobile phone-based apps will make it easier for farmers to identify diseases. Furthermore, plant protection robots moving in the field also need to be able to identify diseases in real time on edge devices. In order to tackle the computational limitations and hardware constraints, many methods for compressing models have been proposed, such as knowledge distillation (Hinton et al., 2015), network pruning (He et al., 2018), weight quantization (Courbariaux et al., 2015), and design of lightweight networks (Howard et al., 2017). Li et al. (2016) pruned the characteristic graph with a small L1 norm of the filter by calculating the L1 norm of the filter. Ayinde and Zurada (2018) proposed an efficient technique to prune redundant features along with their connecting feature maps according to their differentiation and based on their relative cosine distances in the feature space. Lin et al. (2019) proposed a filter pruning scheme termed structured sparsity regularization (SSR). The scheme incorporates two different regularizers of structured sparsity into the original objective function of filter pruning, which fully coordinates the global output and local pruning operations to prune filters adaptively. These compression methods can solve the overparameterization of large neural networks and reduce the computational cost.

Using smart devices to identify crop diseases in the field is a promising approach (Li et al., 2020). Nalepa et al. (2020) tackle the problem of large memory requirements of DCNN in HSI classification and segmentation of hyperspectral images and presented quantizing spectral models for the tasks. Currently, most compact models for disease recognition are directly trained *via* lightweight networks. Tahir et al. (2021) presented disease recognition from the apple leaves based on InceptionV3 and achieved an accuracy of 97% on PlantVillage. Chen et al. (2021) used MobileNet-V2 as the backbone model and combined transfer learning to create a disease identification network for rice identification, with an accuracy rate of 98.48%, which can be deployed on mobile devices. Li et al. (2020) proposed a solanaceae disease recognition model based on SE-Inception, deployed on android phone. The accuracy of the model on the self-built dataset and the PlantVillge reached 98.29% and 99.27%, respectively, and the model sizes were 14.68 MB and 14.8 MB, respectively. Noon et al. (2021) used eight versions of EfficientNet and two versions of MobileNet to train the lightweight models for cotton disease identification, where the EfficientNet-B0 model had the best generalization ability and fastest inference ability. Liu and Wang (2020) used the MobileNetV2-YOLOV3 model to identify tomato diseases and achieved low memory, low latency, high recognition accuracy and high recognition speed. However, according to the information given in the work of Huang (Huang et al., 2017), the deeper the network is, the more effective the training is, and better results can be obtained. Therefore, we can expect that the compressed model will work better than the aforementioned lightweight networks on limited-resource devices.

Currently, more research focuses on improving the accuracy of deep learning models, and less attention is paid to the efficiency of model inference. In this study, when studying disease identification of cotton, we take into account the accuracy, speed of the model, and especially the deployability of the model on edge/mobile devices. We employ a simple but efficient approach of model pruning to compress the high-parameters networks. The γ coefficient in the BN layer is used as the scaling factor for network slimming and the importance of the channel is judged according to γ. In fact, the redundant channels with a small γ value in the disease identification network will be pruned. The well-known networks such as VGG16, ResNet164 and DenseNet40 are selected to train and compress. In order to improve the accuracy of models over our cotton disease dataset, we introduce transfer learning. Combining transfer learning and model compression: 1) compression after transfer learning, and 2) reverse the order. We carry out the experiments to evaluate our methods, and the results indicate that the compressed model can significantly reduce parameters and save time while maintaining the accuracy. Our methods realize the goal of creating a fast and efficient model for the identification of cotton diseases deployed on edge/mobile devices and meet the needs of intelligent agriculture.

## Materials and methods

### Image collection and augmentation

The datasets used in this study include the open plant disease dataset PlantVillage and our self-built cotton disease dataset (SCDD). The images in PlantVillage are taken indoors, with standard photography and simple backgrounds. PlantVillage contains 14 kinds of plants (Apple, blueberry, cherry, corn, grape, orange, peach, bell pepper, potato, raspberry, soybean, pumpkin, strawberry) with 54,306 images of plant disease leaves in total, which falls into 14 kinds of healthy leaves and 24 kinds of disease leaves. The more details of PlantVillage please refer to the work of Hughes (Hughes and Salathé, 2015). Here we only introduce image collection and image preprocessing of SCDD.

**Figure 1:**
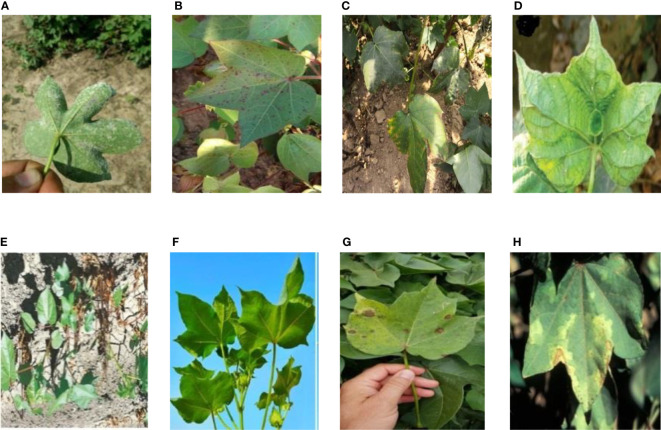
8 Disease images of cotton set: **(A)** Areolate mildew, **(B)** Bacterial blight, **(C)** Brown spot, **(D)** Curl virus, **(E)** Fusarium wilt, **(F)** Healthy, **(G)** Target spot, **(H)** Verticillium wilt.

The cotton disease image set contains images collected from the Internet and taken from the fields. All images are resized to 32×32. A total of 8 types of image samples of cotton were collected, including 7 kinds of the diseases (areolate mildew, bacterial blight, curl virus, fusarium wilt, target spot, verticillium wilt and brown spot) and the healthy leaves. Some of the samples are shown in **Figure 1**.

**Figure 2:**
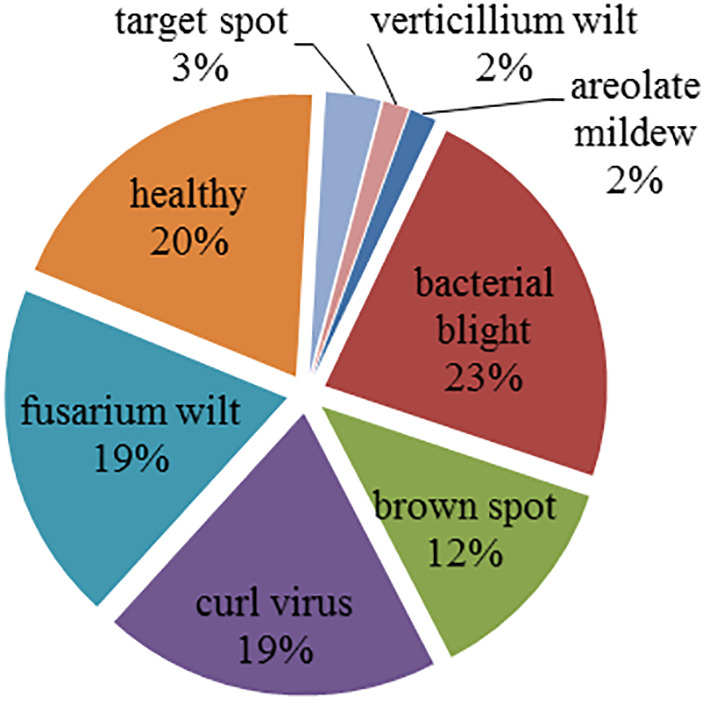
Samples distribution.

**Figure 2** gives the image distribution in the cotton disease image set. It can be seen that the sample distribution of the image set is imbalanced. In detail, the image set contains 34 areolate mildew, 499 bacterial blight, 264 brown spot, 418 curl virus, 419 fusarium wilt, 58 target spot, 34 verticillium wilt, and 425 healthy leaves.

**Figure 3:**
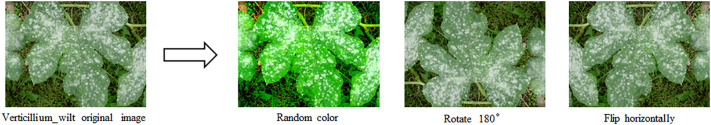
Data augmentation operation.

The imbalance of quantity among different classes means that training of model becomes much trickier as typical accuracy is no longer a reliable metric for measuring the performance of the model. Even if the overall accuracy of the obtained classification model meets the requirements, the accuracy may not be high or even be unpredictable for minority classes. To handle the problem of imbalance classes, we take image augmentation to expand the minority classes including areolate mildew, target spot and verticillium wilt. The approaches contain rotation, random color, and horizontal flip. The examples of the augmented image are shown in **Figure 3**. After the augmentation, the final dataset is called SCDD which consists of 170 areolate mildew, 499 bacterial blight, 264 brown spot, 418 curl virus, 419 fusarium wilt, 357 target spot, 170 verticillium wilt, and 425 healthy leaves.

SCDD is divided into training set and testing set according to the ratio of 80% and 20% (Mohanty et al., 2016), with 2,181 samples in the training set and 542 samples in the testing set. Similarly, PlantVillage is also divided into training set and testing set according to the ratio of 80% and 20%, with 43445 samples in the training set and 10861 samples in the testing set.

### DCNN architectures

In this study, VGG16, ResNet164 and DenseNet40 are selected as the original networks for disease recognition. They have been intensively studied and observed to have good performance for plant disease classification (Bhatt et al., 2017; Ferentinos, 2018; Too et al., 2018; Kundu et al., 2020; Mi et al., 2020).

**Figure 4:**
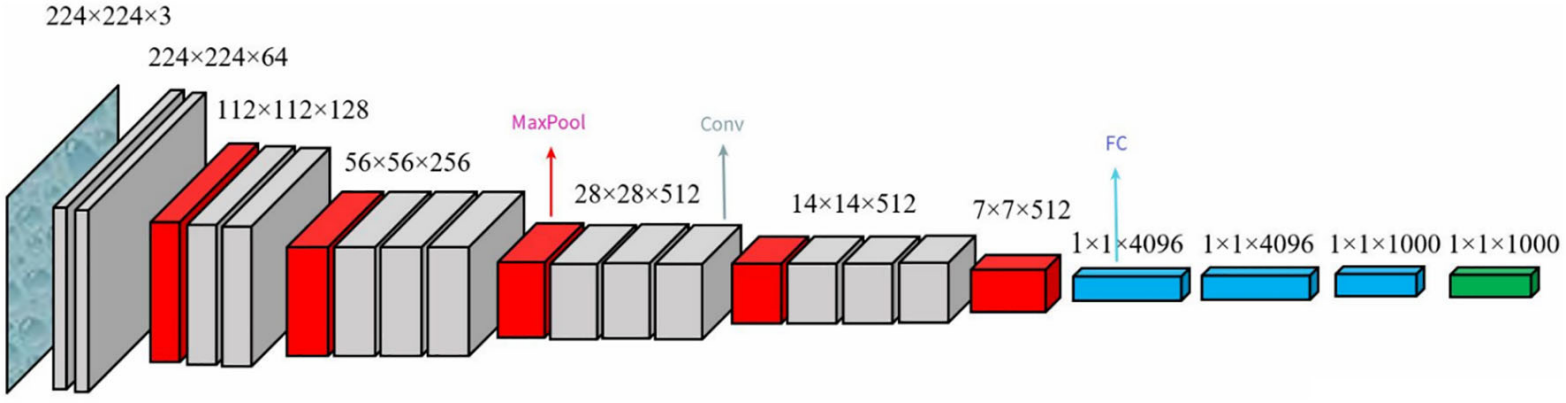
The schematic architecture of VGG.

In 2014, VGG Lab proposed the VGG model (Simonyan and Zisserman, 2014), the schematic architecture of which is shown in **Figure 4**. The classical VGG16 consists of 13 convolutional layers and 3 fully connected layers, using the ReLU function as the activation function with a simple structure. The network uniformly uses 3×3 convolution kernels and 2×2 max-pooling size. Compared with AlexNet, VGG reduces the number of parameters, saves training time, increases the discriminative power of the function, and makes the network more robust by using 3×3 convolutional kernels instead of large-scale convolutional kernels. The VGG16 has 138 million parameters, and the model size is over 500 MB. The VGG16 model used in this study is a variation of the original VGG, which is taken from https://github.com/szagoruyko/cifar.torch . It is smaller than the classical VGG16 model, and the final classification layer of the model is modified to meet the requirement of 8-classified disease images of SCDD.

**Figure 5:**
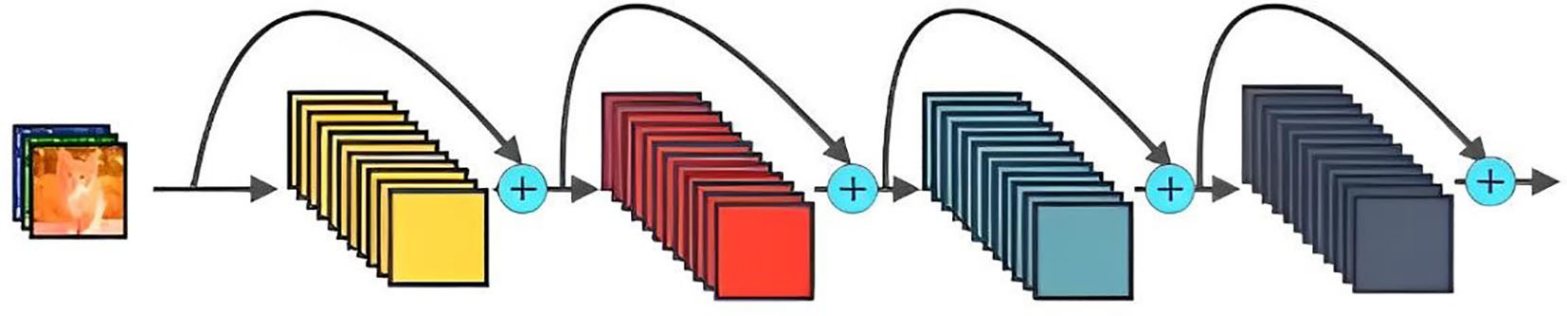
The schematic architecture of ResNet.

The ResNet network was proposed by He et al. (He et al., 2016). **Figure 5** give its schematic architecture. The most significant feature of ResNet is the introduction of residual module, which solves the problems of difficult training and slow convergence caused by the deepening of the number of layers. The ResNet network discards the Dropout mechanism and uses Batch Normalization instead to speed up training. The classical ResNet-152 has 60 million parameters and requires 230MB of storage space. This study uses a framework of 164-layer pre-activated pre-ResNet (He et al., 2016) with a bottleneck structure and modifies the network structure of the model classification layer to apply to the classification of eight crop disease images.

**Figure 6:**
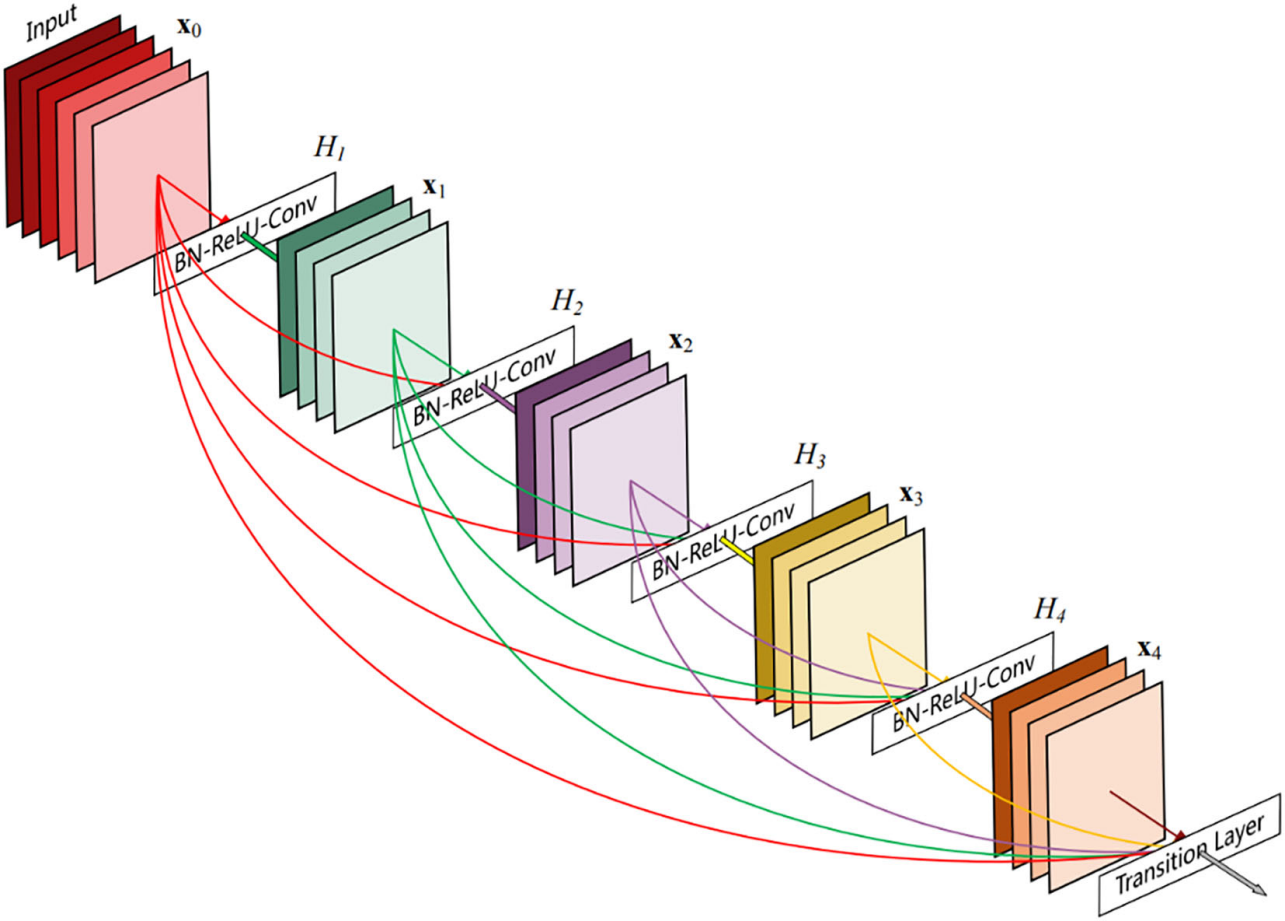
The schematic architecture of DenseNet.

The DenseNet network was proposed by Huang et al. (Huang et al., 2017). **Figure 6** shows its schematic architecture. Compared with ResNet, it has fewer parameters, strengthens feature reuse, aggregates different levels of features using concatenate, and has a regularization effect. The DenseNet is mainly composed of alternate connections between Dense Block and Transition layers. In the core structure Dense Block, the input of the current layer is the union of the output feature maps of all previous layers, and the output feature maps of the current layer are passed to all subsequent layers. The utilization rate of feature maps of each layer is improved, and the problem of gradient disappearance or explosion is effectively solved. Transition layers are placed behind the Dense Block to reduce the number of channels in the feature map and simplify the calculation. This paper constructs a DenseNet40 network with only 40 layers and modifies the output of the network classification layer to 8 classifications.

### Pruning algorithm

The model training is the process of learning the data distribution. The update of parameters causes the input data of each layer to change constantly, so the network needs to change constantly to adapt to this new data distribution, which leads to slow convergence. To solve this problem, Ioffe and Szegedy (2015) proposed the concept of the Batch Normalization (BN) layer, which is also a network layer like the convolutional layer. The BN layer normalizes the input data, and the processed output value is shown in formula (1):


(1) $${\overset{⌢}{x}}_{i}=\frac{x_{i}-\mu_{B}}{\sqrt{\delta^{2}+\epsilon }}$$

(2) $$\mu_{B}=\frac{1}{\text{m}}\sum_{i=1}^{m}x_{i}$$

(3) $$\delta_{B}^{2}=\frac{1}{m}\sum_{i=1}^{m}(x_{i}-\mu_{B}{)}^{2}$$

Where*x*_*i*_ is the input sample value, ${\overset{⌢}{x}}_{i}$ is the normalized sample value,*μ*_*B*_ and*δ*_*B*_ are the mean and variance, *ϵ* is a very small value, which is set to prevent the denominator from being zero and can be taken as10^−8^ , *m* is the number of samples in a single batch.

In order to prevent the generalization performance of the network from being weakened after batch normalization, two learnable parameters *γ* and *β* are introduced:


(4) $$y_{i}=\gamma_{i}{\overset{⌢}{x}}_{i}+\beta_{i}$$

Where *y*_*i*_ is the output of BN layer, *γ*_*i*_ and *β*_*i*_ are the scaling factor and offset function corresponding to the activation channel respectively.

We adopt a simple but efficient method that utilized the *γ* coefficient as the scaling factor of network slimming (Liu et al., 2017). The importance of the channel is judged according to the size of *γ* to prune redundant parameters in the disease identification network. Generally, the model structure adopts the convolution layer + BN layer so that each channel will correspond to one *γ* value. The value of *γ* represents the importance of the channel. The larger the *γ* value, the greater the contribution of the corresponding channel to the network. Conversely, the smaller the contribution. Therefore, the channel with small *γ* value can be pruned to simplify the network scale. In normal training, the weight of the BN layer of the model is generally larger than zero. If the convolution channel corresponding to the weight of the BN layer is directly pruned, it will have a significant impact on the model. Therefore, we need to perform sparse training which is to add the regularization loss of the BN parameter to the original loss function to make the BN parameter tend to zero. Formula (5) is the objective function with the BN regularization loss function.


(5) L=∑l(f(x,W),y)+λ∑g(γ)

Where*x* is the training input, *y* is the training target, *W* is the trainable weight, the first sum term is the original loss function of the convolutional neural network, and *g*(.) is the sparse induced penalty function on the scaling factor. In this study, we chose the *L_1_* norm,*g*(*s*)=|*s*| , which is widely used to achieve parameter sparsity (Liu et al., 2017). *λ* is the balance factor of these two sum terms, and *L* is the loss function during sparse training.

**Figure 7:**
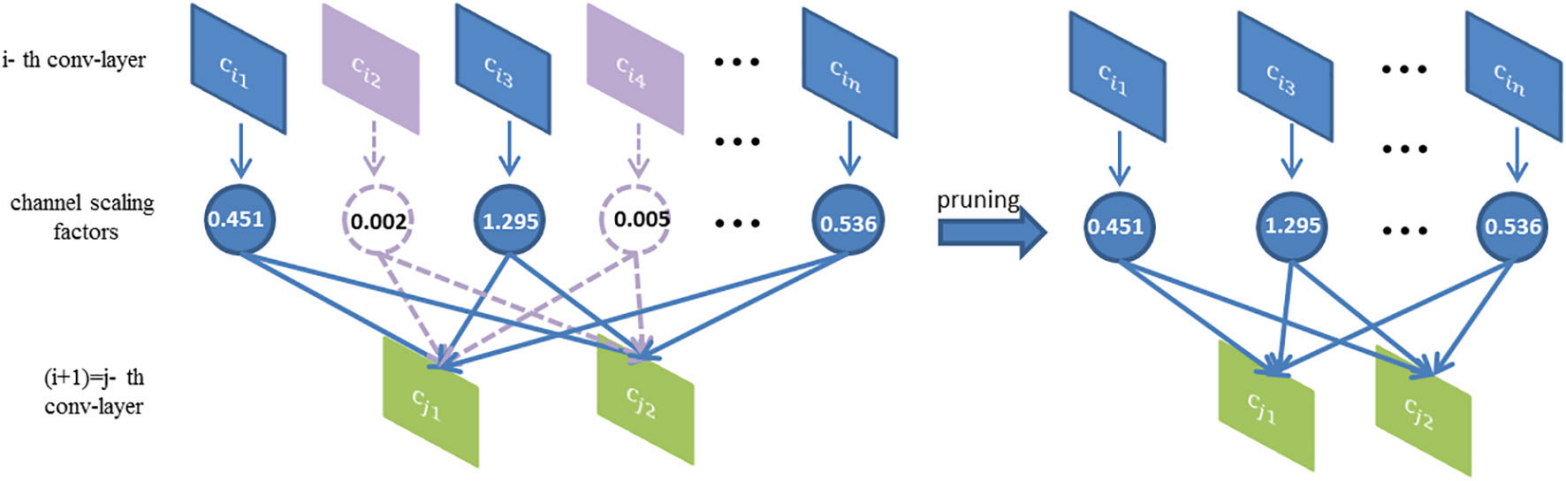
Principle of pruning.

The channels are pruned according to the importance evaluation factor *γ*. Its essence is to prune all the input-output relations connected to it. As shown in **Figure 7**, the channel corresponding to the smaller value of the scaling factor (purple) is pruned, that is, all branches connected to it are pruned (left), and the channel corresponding to the bigger value of the scaling factor is kept (blue). After pruning, a small and efficient network is obtained (right).

**Figure 8:**
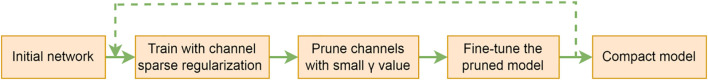
Flowchart of pruning.

The pruning steps are shown in **Figure 8**. First, an original network with a complex structure and many parameters are trained normally to obtain the baseline model. The original network is then trained with sparse regularization so that most of the scaling factors *γ* of the network are close to zero to obtain the sparse model. Then the *γ* values of the obtained BN layers are sorted, and the channels are pruned according to a global threshold across all layers. The global threshold is defined as some percentile of all scaling factor values. If the pruning rate is set to 80%, the channels of 80% with small *γ* values will be pruned. Finally, the pruned compact network is fine-tuned so that the remaining weights are used for training a compact model with comparable performance to the baseline model. Fine-tuning is to retrain the pruned model over SCDD.

### Transfer learning and compression

**Figure 9:**
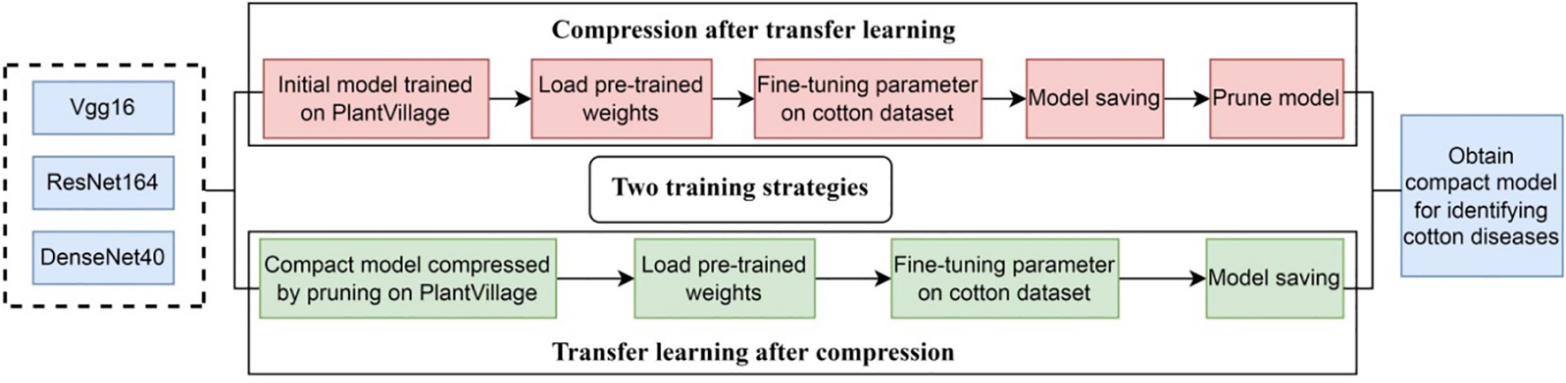
Combination of model pruning and transfer learning.

Transfer learning is the improvement of learning a new task through the transfer of knowledge from a related task that has already been learned (Weiss et al., 2016; Zhuang et al., 2020). In transfer learning, a base network is first trained on the source domain, and then the learned features are transferred to a second target network to be trained on target domain. This process will tend to work if the features are general, meaning suitable to both base and target tasks, instead of specific to the base task. In general, the source domain contains plenty of trainable samples, while the target domain does not. It is a popular approach in deep learning where pre-trained models are used as the starting point on computer vision tasks. Our goal is to train a lightweight network and classify cotton diseases. However, SCDD is too small, if training the network directly on it may lead to the problems such as low recognition accuracy or overfitting. Transfer learning can solve these problems very well (Ghazi et al., 2017; Chen et al., 2020; Wenchao and Zhi, 2022). The key to transfer learning is to find out the similarities between the source domain and the target domain (Gao and Mosalam, 2018). Thus, we select PlantVillage as the source domain and SCDD as the target domain due to both being plant diseases recognition tasks and the former having more disease categories and a lot of data. We train the networks over the source domain as the pre-trained models and then fine-tune those models over the target domain. Model compression and transfer learning play different roles in our study. The goal of the former is to provide models with a small size that can be deployed at edge/mobile devices, while the goal of the latter is to improve accuracy. Considering both goals, we combine both techniques in our methods. As shown in **Figure 9**, two strategies are proposed: (1) compression after transfer learning, and (2) transfer learning after compression. In the first case: (1) The original models are trained over PlantVillage as the pre-trained models. (2) The pre-trained models are fine-tuned over SCDD by the transfer learning. (3) Finally, the fine-tuned models are pruned to obtain the compact models. In the second case, the compression of the original models is first carried out over PlantVillage, and then the compressed models as the pre-trained models are fine-tuned over SCDD.

### Model evaluation index

Accuracy is an important index for evaluating classification models, and the larger it is, the better the performance of the model. Model parameters and floating point of operations (FLOPs) are two important indicators for deployment on small equipment. The computation resource of the mobile/edge devices is very limited. If the model is too complex, the application will get stuck and the response not be in time. In order to meet the hardware conditions of the mobile/edge devices, the classification accuracy of model should be high, and FLOPs and parameters of model should be small.

The model classification accuracy is the number of correct model predictions in a batch of data as a percentage of the total number of data in the batch.


(6) $$Accuracy=\frac{TP+TN}{TP+FP+TN+FN}\times 100\%$$

Where *TP* is correctly predicted positive values, *FP* is incorrectly predicted positive values, *TN* is correctly predicted negative values and *FN* is incorrectly predicted negative values.

The structure of convolutional neural networks mainly includes convolution layers, activation layers, pooling layers and full connection layers, and core layers are convolutional layers. Convolutional layers are mainly used to extract image features in neural networks. Pooling layers are used to compress the feature map. The main pooling methods include average pooling and max pooling (Boureau et al., 2011), that is, the average or max value of specific features in a certain region is kept during the pooling operation. Its goal is to save helpful information while reducing network parameters. Full connection layers classify and integrate the highly abstracted characteristics produced by convolution layers. Pooling layers have no associated parameters in convolutional neural networks. The number of parameters for the convolutional layers is calculated in formula (7):


(7) $$Params=C_{o}*(K_{W}*K_{h}*C_{i}+1)$$

Where*C*_*o*_ is the number of output channels,*K*_*w*_ and*K*_*h*_ are the width and height of the convolution kernel respectively,*C*_*i*_ is the number of input channels, and +1 is the bias unit.

The number of parameters for the fully connected layer is calculated in formula (8):


(8) Params=(I+1)∗O

Where *I* is the number of input neurons, and *O* is the number of output neurons. *FLOPs* are used to measure the complexity of a model, that is, computation. The *FLOPs* of convolutional layers are calculated by formula (9):


(9) $$FLOPs=2*H*W(C_{i}*K^{2}+1)*C_{o}$$

Where*C*_*i*_ is the number of input channels, *K* is the size of the convolution kernel, *H* and *W* are the height and width of the output feature map, and*C*_*o*_ is the number of output channels.

The *FLOPs* of full connection layer is calculated by the formula (10):


(10) FLOPs=(2×I−1)×O

Where I is the number of input neurons, and O is the number of output neurons.

## Results and discussion

### Experimental setup

For each model, we set the batch size of training as 64 and the batch size of testing as 256, and the training epoch as 100. We use stochastic gradient descent (SGD) as the optimization method. The initial learning rate is 0.001 for VGG16 and 0.1 for ResNet164 and DenseNet40. The learning rate is multiplied by 0.1 at 50% and 75% epochs. The development environment is as follows: the operation system is Ubuntu 18.04.6 LTS 64-bit, the programming language is python 3.6, the deep learning frameworks are pytorch 1.3, and the IDE is pycharm 2020.3.5. The hardware environment of the computer for training is configured as below: 64GB memory, Intel^®^ Xeon(R) Silver 4110 CPU @ 2.10GHz x64 processor, NVIDIA Tesla K40 GPU. In the following sections, we randomly form 5 sets of train set and test set adhering to rule of Section 2.1 and depending on the experimental setup, train 5 sets of models of VGG, ResNet and DenseNet for the best results (accuracy) and statistical analysis.

### Performance test results over PlantVillage

First, we evaluate the performances of original VGG16, ResNet164 and DenseNet40 and their compressed versions over PlantVillage. In the experiments, the pruning rate is set to 80%, and the best results out of 5 experiments are given in **Table 1**. It is shown that the parameters of VGG16, ResNet164 and DenseNet40 are compressed to 0.32M, 0.37M and 0.27M, respectively, and their FLOPs are compressed to 0.01G, 0.05G and 0.1G respectively. Meanwhile, the recognition accuracies of all the models before and after pruning are nearly the same. DenseNet40-80% even slightly surpasses its original version. This shows that the presented pruning algorithm can not only reduce the model’s size greatly, but also keep high accuracy.

**Table 1:** Comparison before and after compression over PlantVillage.

Model	Accuracy	Parameters/M	Parameters Pruned	FLOPs/G	FLOPs Pruned	Size/MB	Size Pruned
VGG16	96.76%	14.74	–	0.31	–	118	–
VGG16-80%	97.46%	0.32	97.83%	0.01	96.77%	2.6	97.8%
ResNet164	99.55%	1.72	–	0.26	–	14.1	–
ResNet164-80%	99.12%	0.37	78.49%	0.05	80.77%	3.3	76.60%
DenseNet40	99.62%	1.08	–	0.29	–	8.8	–
DenseNet40-80%	99.68%	0.27	75.00%	0.1	65.52%	2.3	73.86%

Results on PlantVilage. *-80% represents the pruning rate of 80%. The pruned models in the table are all fine-tuned models.

### Compression after transfer learning

We adopt the original networks trained over PlantVillage as pre-trained networks, and perform transfer learning over SCDD to get the baseline models for identifying cotton disease. The baseline models are then compressed using the presented pruning algorithm, and the pruned models are retrained again using fine-tuning to compensate for the accuracy lost during the pruning phase. In the experiments, the pruning rates are set to 70% and 80%, respectively, and the epoch of all models is set to 100. The experimental results are shown in **Table 2**.

**Table 2:** Comparison of parameters of cotton disease identification model before and after compression.

Model	Accuracy	Parameters/M	Parameters Pruned	FLOPs/G	FLOPs Pruned	Size/MB	Size Pruned
VGG16	87.27%	14.72	–	0.31	–		–
T-VGG16	92.80%	14.72	–	0.31	–	117.8	–
T-VGG16-70%	91.14%	0.86	94.15%	0.03	90.32%	6.9	94.14%
T-VGG16-80%	89.48%	0.30	97.96%	0.01	96.77%	2.4	97.96%
ResNet164	82.29%	1.71	–	0.26	–		–
T-ResNet164	95.57%	1.71	–	0.26	–	14.0	–
T-ResNet164-70%	94.28%	0.65	61.98%	0.12	53.84%	5.5	60.71%
T-ResNet164-80%	94.65%	0.43	74.85%	0.08	69.23%	3.7	73.57%
DenseNet40	89.30%	1.07	–	0.29	–	8.7	–
T-DenseNet40	96.49%	1.07	–	0.29	–	8.7	–
T-DenseNet40-70%	96.31%	0.36	66.35%	0.13	55.17%	3.0	65.51%
T-DenseNet40-80%	96.86%	0.26	75.70%	0.10	65.52%	2.2	74.71%

Results over SCDD. T-* denotes transfer learning before compression. *-70% and *-80% represent the fine-tuned models with the pruning rates of 70% and 80%, respectively.

We first train the original VGG16, ResNet164, and DenseNet40 from scratch over SCDD to test the performance of the three networks, which achieve 87.27%, 82.29%, and 89.30% accuracy, respectively. To improve the accuracy of the models, we then carry out transfer learning over SCDD to obtain baseline models T-VGG16, T-ResNet164 and T-DenseNet40. By transfer learning, the accuracies of T-VGG16, T-ResNet164 and T-DenseNet40 are improved by 5.53%, 13.28%, and 7.19% compared with their original versions, respectively. The T-Densenet40, due to its own structure with the advantage of feature reuse, coupled with the strategy of transfer learning, has the best recognition effect among the three baseline models. It can be seen from **Table 2** that the accuracy of the models with 80% pruning rate, in summary, are similar with the models with 70% pruning rate. However, the numbers of parameters of the latter are roughly half that of the former. After pruning, the T-VGG16-80% has an accuracy of 89.48% over the testing set. Compared to its baseline model, it only loses 3.32% accuracy, but its parameters are reduced from 14.72M to 0.30M, and FLOPs are reduced from 0.31G to 0.01G. Its actual pruning ratio is the highest. T-ResNet164-80% has an accuracy of 94.65% over the testing set and loses 0.92% of the accuracy compared to its baseline model, which loses less accuracy than T-VGG16-80%. The actual pruning ratios of T-ResNet164-80% and T-DenseNet40-80% are not as significant as T-VGG16-80%. T-DenseNet40-80% has an accuracy of 96.86% which is higher than its baseline model, increased by 0.37%. Since the original DenseNet40 has fewer parameters, the pruned T-DenseNet40-80% has the smallest parameters of 0.26M. Among the three compressed models, the T-DenseNet40-80% has the highest accuracy and the smallest parameters and size, and the T-VGG16-80% has the smallest FLOPs and the fastest speed. The findings indicate that the pruned models require substantially fewer parameters and FLOPs. Therefore, using the pruning algorithm to compress the cotton disease identification model achieve our expected result: less model size and running faster.

**Figure 10:**
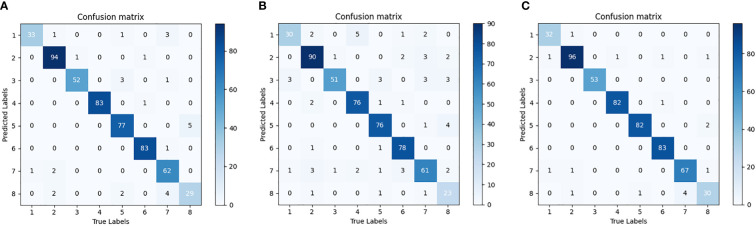
Confusion matrix of the pruned model. **(A)** T-VGG16-80%, **(B)** T-ResNet164-80%, **(C)** T-DenseNet40-80%. Areolate mildew 1, bacterial blight 2, brown spot 3, curl virus 4, fusarium wilt 5, healthy 6, target spot 7, verticillium wilt 8.

In order to further verify the performance of the compact model, **Figure 10** shows the confusion matrices of the three compact models with 80% pruning rate over the cotton testing set, respectively. The value at the diagonal shows the number of samples correctly predicted. The testing set of SCDD has a total of 542 samples. The confusion matrix indicates the recognition ability of each compact model over the set. T-VGG16-80% has the most errors among the three models. The top two diseases misclassified by it are verticillium wilt (11 out of 34) and target spot (10 out of 71). Target spot is the most likely to be confused with other diseases by T-VGG16-80%, the rest of 7 classes all have been mistakenly identified as it. T-DenseNet40-80% has minimal errors among the three models. The highest error rate is still verticillium wilt (4 out of 34), and the second error rate is areolate mildew (2 out of 34). Compared with T-VGG16-80%, the errors of verticillium wilt and target spot misclassified by T-DenseNet40-80% are greatly reduced. This shows that the network has better discrimination ability. For T-DenseNet40-80%, verticillium wilt is the most likely to be confused with the rest. There are 6 samples of 3 categories being misclassified as it. The performance of T-ResNet164-80% is between T-DenseNet40-80% and T-VGG16-80%. The misclassified samples are uniformly distributed in the confusion matrix of each model, indicating that each of them has no bias over SCDD.

### Transfer learning after compression

In this case, the pruned models, VGG16-80%, ResNet164-80% and DenseNet40-80% over PlantVillage as pre-trained models and fine-tuned over SCDD to obtain compact models, denoted as VGG16-80%-T, ResNet164-80%-T and DenseNet40-80%-T.

**Figure 11:**
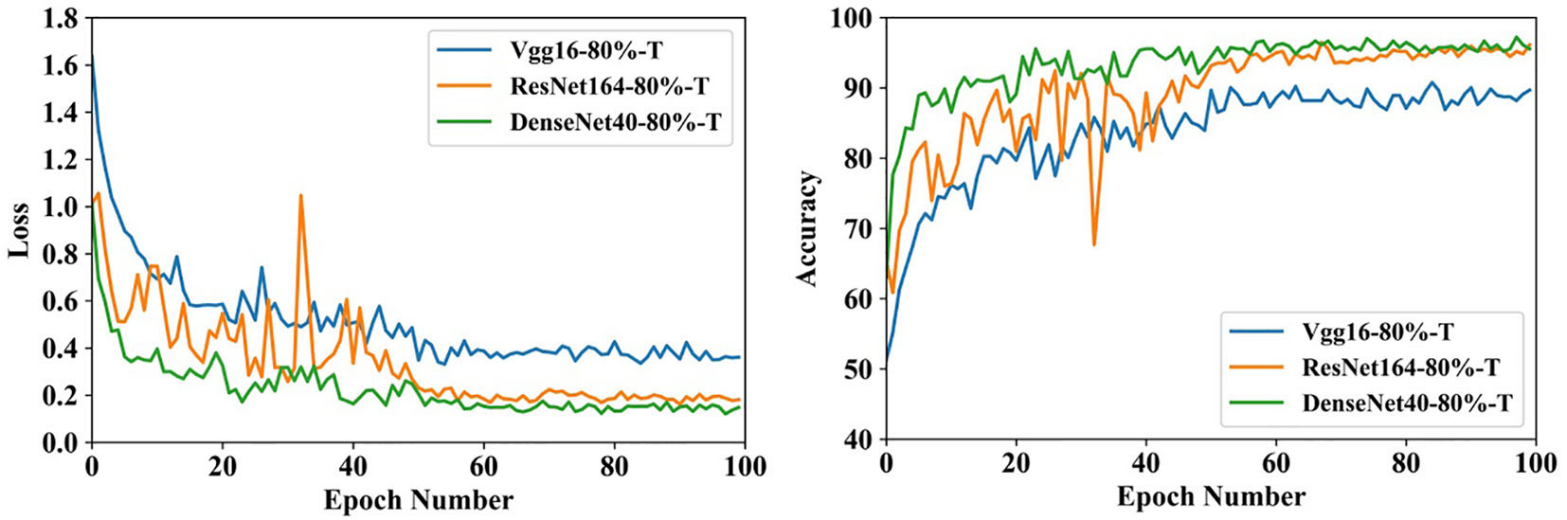
Training process of fine-tuning over SCDD in case of transfer learning after compression.

**Figure 11** shows the training process that three compact models fine-tune the parameters over SCDD. The initial accuracies of the three models exceed 50%, which shows that the target domain and source domain have a lot in common. Furthermore, thanks to small sizes, all the models converge very fast within 60 epochs.

The best results out of 5 experiments are shown in **Table 3**. The sizes of parameters and FLOPs of VGG16-80%-T and DenseNet40-80%-T are the same as T-VGG16-80% and T-DenseNet40-80%, respectively. The accuracies of them are 90.77% and 97.23%, which are 1.29% and 0.37% higher than T-VGG16-80% and T-DenseNet40-80%, respectively. The parameters and FLOPs of ResNet164-80%-T are 0.36M and 0.05G, respectively, smaller than T-ResNet164-80%. Its accuracy is 96.31%, with an improvement of 1.66%. The accuracy of DenseNet40-80%-T is still the highest, showing that DenseNet40-80%-T is more suitable for the cotton disease recognition. Compared with their baseline models, VGG16-80%-T loses 2.03% accuracy, which is less than T-VGG16-80%, and ResNet164-80%-T and DenseNet40-80%-T both improve the accuracy by 0.74%.

**Table 3:** Results of transfer learning after compression.

Model	Accuracy	Parameters/M	FLOPs/G	Size/MB
VGG16-80%-T	90.77%	0.30	0.01	2.4
ResNet164-80%-T	96.31%	0.36	0.05	3.2
DenseNet40-80%-T	97.23%	0.26	0.1	2.2

Results over SCDD. *-T denotes transfer learning after compression.

Usually, accuracy may not fully evaluate the model, especially in the case of imbalanced sample distribution. **Table 4** gives the other performance indicators, including Precision, Recall and F1-score. It can be seen that the performance of the compressed models remains stable when we adopt image augmentation.

**Table 4:** Performance of compressed models.

Network	Accuracy	Precision	Recall	F1-score
Vgg16-80%-T	90.77%	89.56%	89.41%	89.45%
ResNet164-80%-T	96.31%	95.54%	96.15%	95.81%
DenseNet40-80%-T	97.23%	96.64%	97.24%	96.92%

The above results indicate that compared with compression after transfer learning, transfer learning after compression has two advantages: (1) higher accuracy, and (2) faster training speed. Among the three models, DenseNet40-80%-T is the best, so we select it as the winner to participate in the follow-up experiments.

### Comparing two strategies using the t-test

By comparing **Table 2** with **Table 3**, it can be seen that the accuracies of transfer learning after compression (strategy 2) are higher than that of compression after transfer learning (strategy 1). Our further statistical analysis supports the claim. **Table 5** gives the details of 2 sets of 5 models with respect to 2 strategies. We perform independent sample t-tests on the accuracies to test the significance of the differences between them. Levene’s test is used to examine the homogeneity of variance. When P>0.05, the variance is homogeneous, and when P ≤ 0.05, the variance is not homogeneous. The p-value of the t-test is employed to determine the significance of the mean of the accuracy. The results of the t-test are shown in **Table 6**. For the VGG16, the variance is homogeneous. The difference between strategy 1 and strategy 2 is significant (P = 0.039< 0.05). Since the mean of strategy 1 is 88.67% and that of strategy 2 is 89.74%, strategy 2 is better than strategy 1. For the ResNet164, the variance is homogeneous. The difference between strategy 1 and strategy 2 is significant (P =0.0< 0.0001). Since the mean of strategy 1 is 94.44% and that of strategy 2 is 96.11%, strategy 2 is better than strategy 1. For the DenseNet40, the variance is homogeneous. The difference between strategy 1 and strategy 2 is significant (P=0.045<0.05). Since the mean of strategy 1 is 96.62% and that of strategy 2 is 96.97%, strategy 2 is better than strategy 1. The above analyses indicate that, for VGG, ResNet and DenseNet, strategy 2 is a better choice than strategy 1.

**Table 5:** Accuracy of 2 sets of 5 models w.r.t. 2 strategies.

Strategies	VGG16	ResNet164	DenseNet40
1	88.93%	94.28%	96.68%
	87.45%	94.37%	96.86%
	88.75%	94.46%	96.32%
	89.48%	94.65%	96.68%
	88.75%	94.46%	96.56%
2	89.48%	96.13%	97.05%
	90.77%	96.31%	97.23%
	89.11%	96.31%	96.53%
	89.67%	96.25%	96.98%
	89.67%	95.57%	97.05%

**Table 6:** Independent sample t-tests on accuracy w.r.t. 2 strategies.

Levene’s Test forEquality of Vanances				t-test for Equality of Means				
		F	Sig.	t	df	Sig(2-tailed)	Mean Difference	Std. ErrorDifference
VGG16	Equality Vanances assumed	0.069	0.800	-2.463	8	0.039	-1.06800	0.43359
	Equality Vanances not assumed			-2.463	7.740	0.040	-1.06800	0.43359
ResNet164	Equality Vanances assumed	1.622	0.239	-10.931	8	0.000	-1.67000	0.15278
	Equality Vanances not assumed			-10.931	5.484	0.000	-1.67000	0.15278
DenseNet40	Equality Vanances assumed	0.111	0.748	-2.367	8	0.045	-0.34800	0.14705
	Equality Vanances not assumed			-2.367	7.466	0.048	-0.34800	0.14705

### Comparison with lightweight networks

It is a very popular method in plant disease recognition that directly trains a light-weight network as a classifier (Liu and Wang, 2020; Tahir et al., 2021; Chen et al., 2021). We carry out comparative experiments over SCDD with some popular light-weight networks, including MobileNetV2 (Sandler et al., 2018), MobileNetV3 (Howard et al., 2019), ShuffleNetV2_x_0 (Ma et al., 2018), EfficientNet-B0 (Tan and Le, 2019) and EfficientNetV2-S (Tan and Le, 2021). These networks are fine-tuned using transfer learning. The results are described in **Table 7**. It can be seen from the table that DenseNet40-80%-T has the highest accuracy and the smallest parameters and model sizes among these models. Our compressed model defeats the lightweight networks in the comparison. This result shows that, after proper compression and transfer learning, the large models usually have better performance than the lightweight networks and can meet the small size requirements of mobile/edge applications while retaining high accuracy.

**Table 7:** Performance comparison of light-weight models and our compressed model.

Network	Accuracy	Parameters/M	FLOPs/G	Size/MB
MobileNetV2	92.15%	2.23	0.007	18.1
MobileNetV3	81.37%	1.53	0.002	18.5
ShuffleNetV2_x1_0	90.21%	1.26	0.003	10.3
EfficientNet-B0	56.13%	4.56	0.009	32.4
EfficientNetV2-S	90.01%	20.19	0.06	162.4
DenseNet40-80%-T	**97.23%**	**0.26**	0.1	**2.2**

The meaning of bold values has been described in 1257 line. Compared with several lightweight networks, the accuracy and the smallest parameters and model sizes among these models.

### Developing cotton disease recognition APP based on DenseNet40-80%-T

**Figure 12:**
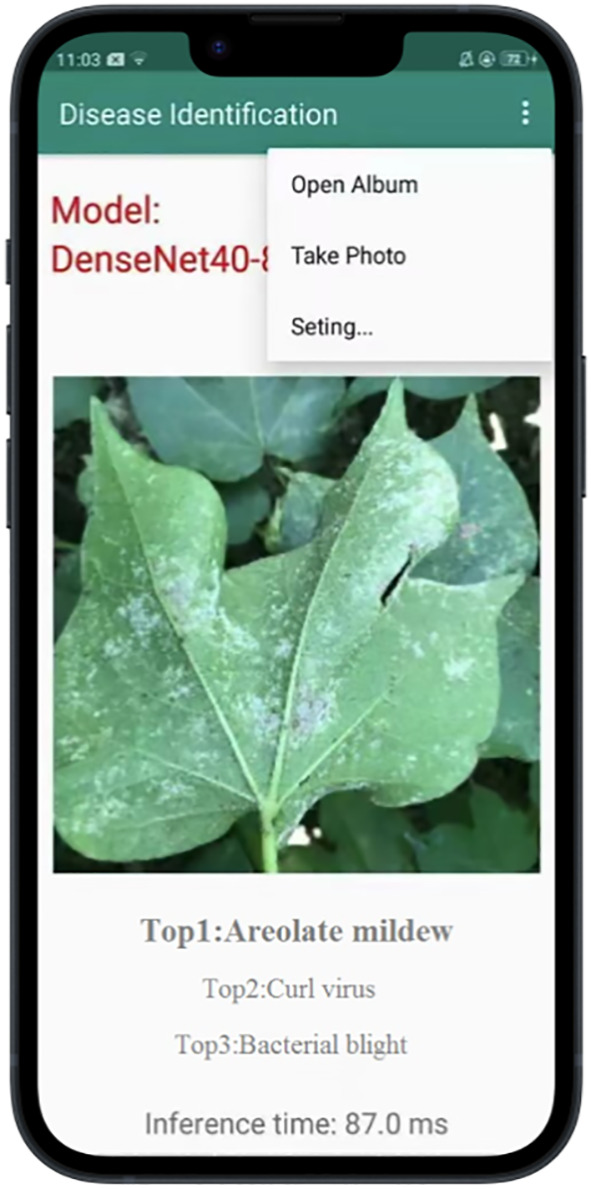
User interface of our APP.

According to the previous results, we employ DenseNet40-80%-T to develop a cotton disease recognition APP based on the Android platform. Our model is deployed locally on the mobile phone. The development software of the APP is Android Studio (https://developer.android.google.cn/). The classification model import process includes: 1) model preparation, 2) model import and parameter modification, and 3) APP installation. The model preparation is mainly to get the nb file and the txt label file on the computer. The model compression is done under the Pytorch framework. The compressed model is deployed *via* paddle-lite under the PaddlePaddle framework (https://www.paddlepaddle.org.cn/ ). We convert the compressed Pytorch model to the Paddle model, and then use Paddle-lite to convert the pd file to the nb file for deployment. The model import and parameter modification are to open the Project view in Android Studio, define variables, initialize the interface, configure the corresponding (build gradle) version of the file, and put the nb file and txt file under app/src/main/assets. We port the installation package to the Android phone by wired means and clicked to install the APP on the Android phone. The user interface of the APP is shown in **Figure 12**. Users can upload a photo of any size by shooting and local uploading. We deploy the APP on OPPO A5 mobile phone. The disease recognition can be carried out in real time, with the average time of a single image being 87ms.

## Conclusion

Early-stage disease identification can reduce crop losses. DCNN have shown good performance in the automation of the disease identification task. However, most DCNN have a large number of parameters and calculations, making them difficult to deploy on mobile/edge devices. At present, most of the core modules of the identification tasks in agricultural applications run on the server side, while mobile/edge devices only play the role of information collection and display results. This model is highly dependent on the communication network and does not work in the region of poor signal coverage. In response to the problem of cotton disease identification in the field, combined with transfer learning, we present a simple but effective pruning algorithm to compress several DCNN networks. The method is to judge the importance of the channel according to γ value and prune the channel with a small γ value. The results are promising that the parameters and FLOPs of the models compressed by the two strategies can be greatly reduced while maintaining the high accuracy of the big models. The DenseNet40-80%-T compressed by the strategy of transfer learning after compression has the smallest size and the highest accuracy among the compressed models, which can be easy to deploy on mobile or edge devices. To further verify the feasibility and validity of the compression strategy, we conduct experiments to compare the compressed model with some famous light-weight models over SCDD. Experimental results demonstrated the DenseNet40-80%-T, even under complex background conditions, the average accuracy reaches 97.23% and both recognition accuracy and model size are superior to other competitors. Finally, we adopt DenseNet40-80%-T as recognition model to develop the APP for cotton disease classification and the result shows that the APP can identify the cotton disease in real time.

## Data availability statement

The original contributions presented in the study are included in the article/supplementary material. Further inquiries can be directed to the corresponding author.

## Author contributions

DZ and QF collected data and designed the experiments. DZ performed the experiment, selected the algorithm, analyzed the data, trained the algorithms, and wrote the manuscript. QF revised the manuscript. JZ and WY gave guidance to this research. All authors contributed to the article and approved the submitted version.
